# The Effect of Nurse‐Led Transitional Care on Rehabilitation Outcomes in Patients With Lumbar Disc Herniation: A Retrospective Cohort Study

**DOI:** 10.1155/nrp/5191926

**Published:** 2026-08-12

**Authors:** Jing Guo, Jian Li, Jianli Cao, Pingping Liu, Hong Wang

**Affiliations:** ^1^ Hainan District People’s Hospital, Wuhai 016030, Inner Mongolia, China

**Keywords:** lumbar disc herniation, nursing, rehabilitation, retrospective study, transitional care

## Abstract

**Objective:**

This study examined whether documented receipt of nurse‐led transitional care (NTC) was associated with postdischarge rehabilitation outcomes in patients with lumbar disc herniation (LDH).

**Methods:**

We conducted a retrospective cohort study of 134 patients with LDH who received conservative treatment between February 2022 and March 2025. Patients were classified as NTC or routine care (RC) according to the documented postdischarge nursing pathway. Outcomes included VAS, ODI, SF‐36, ESCA, and GS at baseline and at 1, 2, and 3 months. Longitudinal changes were evaluated using generalized estimating equations, and 3‐month outcomes were examined with multivariable linear regression adjusted for age, sex, length of hospital stay, follow‐up duration, and the corresponding baseline score. A propensity score‐matched sensitivity analysis was also performed.

**Results:**

Baseline characteristics were comparable between groups. In unadjusted analyses, the NTC cohort showed lower VAS and ODI scores and higher SF‐36, ESCA, and GS scores than the RC cohort at all follow‐up points (all *p* < 0.01). In adjusted longitudinal analyses, group‐by‐time interactions were significant for VAS (*p* = 0.012) and for ODI, SF‐36, ESCA, and GS (all *p* < 0.001). At 3 months, adjusted estimates remained favorable to NTC for all five outcomes (all *p* < 0.001). Findings remained directionally consistent after propensity score matching.

**Conclusion:**

In this retrospective cohort, documented receipt of NTC was associated with better early postdischarge rehabilitation outcomes in patients with LDH. The overall pattern remained robust in adjusted, sensitivity, and propensity score‐matched analyses, although prospective studies are still needed to address residual confounding.

## 1. Introduction

Lumbar disc herniation (LDH) is a common degenerative spinal disorder in which disc material extends beyond its normal anatomical boundaries and often causes low back pain, radiculopathy, and functional limitation [1]. Because LDH frequently affects working‐age adults and is associated with recurrent symptoms, healthcare use, and work impairment, it also imposes a substantial social and economic burden [2, 3].

Conservative treatment remains the initial approach for many patients with LDH and commonly includes medication, physical therapy, exercise‐based rehabilitation, and routine discharge guidance [4, 5]. Postdischarge support, however, is often fragmented. Conventional nursing care tends to center on the inpatient period, whereas early recovery at home may involve inconsistent education, limited follow‐up, and variable adherence to rehabilitation plans. These gaps may shape symptom control, activity modification, and subsequent recovery.

Transitional care has been proposed as a structured approach to bridge this vulnerable period by linking discharge preparation with ongoing home‐ or community‐based support [6–8]. Nurse‐led transitional care (NTC) typically emphasizes individualized planning, patient education, symptom monitoring, and reinforcement of rehabilitation behaviors [9–12]. Although this model has shown benefits in chronic disease management and continuity‐related outcomes, LDH‐specific research has focused more on continuity‐of‐care patterns and conservative rehabilitation than on structured NTC programs that assess multiple rehabilitation domains simultaneously [8, 10, 13, 14].

Against this background, this retrospective cohort study examined whether documented receipt of NTC was associated with pain intensity, disability, health‐related quality of life, self‐care ability, and gait performance during the first 3 months after discharge in patients with LDH.

## 2. Methods

### 2.1. Study Design and Participants

This retrospective cohort study was approved by the hospital ethics committee (Approval No. 1503032021003). Because the study involved secondary analysis of existing clinical records and deidentified follow‐up data, the requirement for individual informed consent was waived. The study was conducted with reference to the 2022 Guideline for Diagnosis, Treatment and Rehabilitation of Lumbar Disc Herniation issued by the Chinese Rehabilitation Medical Association [15]. Medical records from February 2022 to March 2025 were screened.

Inclusion criteria were as follows: diagnosis of LDH, age 20–60 years, VAS score ≥ 4 at admission, receipt of conservative treatment, and availability of baseline and 1, 2, and 3 months follow‐up data.

Exclusion criteria were as follows: surgical indication during the index admission, pregnancy or breastfeeding, severe mental disorder, severe osteoporosis, or major heart, liver, or kidney dysfunction.

### 2.2. Cohort Formation and Data Collection

Patients were retrospectively classified according to the postdischarge nursing pathway documented in the medical record. Records showing enrollment in the structured NTC pathway were assigned to the NTC cohort, whereas records documenting standard discharge education and routine outpatient follow‐up without pathway‐level NTC documentation were assigned to the routine care (RC) cohort. In routine practice, pathway documentation was generated during discharge planning and early follow‐up workflows; however, the available records did not allow reliable reconstruction of whether pathway entry primarily reflected clinician referral, service workflow, patient acceptance, or a combination of these factors. Cohort assignment should therefore be understood as a documentation‐based exposure classification rather than a randomized allocation process. Demographic variables, baseline outcome scores, length of hospital stay, and follow‐up duration were extracted from the electronic record and standardized nursing follow‐up forms. In this study, follow‐up duration denotes the interval from index admission to the last available documented contact in the record system; it reflects the total record‐observation span available for retrospective abstraction rather than the prespecified outcome assessment schedule, which was limited to baseline and 1, 2, and 3 months of time points. Core intervention components are summarized in Table 1.

**TABLE 1:** Core components of the nurse‐led transitional care pathway.

Component	Timing	Frequency	Delivery mode	Core content
Predischarge assessment and individualized plan	Before discharge	Once during index admission	Bedside assessment	Assessment of symptoms, mobility, activity tolerance, home circumstances, and self‐management needs; development of an individualized rehabilitation plan with the treating team.
Discharge education	Before discharge	At least one structured session	Bedside teaching	Teach‐back education covering disease knowledge, medication use, posture and activity precautions, warning symptoms, home exercises, and follow‐up arrangements.
Early postdischarge follow‐up	Within 48–72 h after discharge	At least one contact	Telephone	Early review of symptoms, activity tolerance, medication use, and exercise adherence, with reinforcement of key discharge instructions.
Ongoing follow‐up reinforcement	Early recovery period after discharge	Repeated as documented	Telephone; home visit for selected high‐risk patients	Monitoring of recovery progress, problem solving, exercise progression, and additional support for patients requiring closer follow‐up.
Self‐management support	Throughout follow‐up	Ongoing	Written materials and coaching	SMART‐based goal setting, exercise logbooks, self‐monitoring, and adherence reinforcement.
Multidisciplinary coordination	As needed during transition	As needed	Documentation and referral	Communication of discharge information and rehabilitation recommendations to relevant outpatient or community providers to support continuity of care.

*Note:* The retrospective records captured the overall structure of the pathway and confirmed at least one early postdischarge contact within the NTC pathway, but they did not allow reliable aggregation of the average number of follow‐up contacts, the exact duration or intensity of each contact, or the proportion of patients who received home visits.

### 2.3. Interventions

NTC cohort: Patients in this cohort received a structured transitional care program informed by the Chronic Care Model and Naylor’s Transitional Care Framework [6, 7, 9]. The program was delivered by a registered nurse trained in the hospital’s transitional care workflow, discharge education procedures, exercise instruction, and follow‐up documentation. The intervention included the following components:

1. Predischarge assessment and individualized plan: Before discharge, the nurse assessed symptoms, mobility, activity tolerance, home circumstances, and self‐management needs, then developed an individualized rehabilitation plan in collaboration with the treating physician and rehabilitation staff.
2. Discharge education: Structured bedside education was delivered before discharge using teach‐back methods. Core topics included disease knowledge, medication use, posture and activity precautions, home exercises, warning symptoms, and follow‐up arrangements.
3. Postdischarge follow‐up: A telephone follow‐up was completed within 48–72 h after discharge. High‐risk patients could receive additional contact according to clinical need. Scheduled follow‐up continued during the early recovery period, with emphasis on symptom review, exercise progression, reinforcement of precautions, and review of home‐exercise adherence. Adherence was assessed pragmatically during follow‐up calls through patient self‐report and, when available, review of exercise logbooks. Because aggregate counts of total follow‐up contacts and home‐visit use were not consistently documented in the retrospective records, average contact frequency and the proportion of home visits could not be reported reliably.
4. Self‐management support: Patients received written educational materials, individualized exercise targets, and self‐monitoring guidance. Nurses reinforced adherence to the home program and encouraged gradual goal attainment based on SMART principles.
5. Multidisciplinary coordination: When needed, discharge information and rehabilitation recommendations were communicated to community or outpatient providers to support continuity of care.

RC cohort: Patients in this cohort received the hospital’s routine discharge education and standard outpatient follow‐up without the structured NTC pathway. RC included general education on LDH, conservative management guidance, psychological support, dietary advice, and return‐visit information, consistent with usual conservative care and patient education practices [15–17].

### 2.4. Outcome Measures

Outcome data were extracted at baseline (hospitalization) and at 1, 2, and 3 months of follow‐up. The assessed domains were pain intensity (VAS), disability (ODI), health‐related quality of life (SF‐36), self‐care ability (ESCA), and gait‐related performance (GS). Lower scores indicate better status for VAS and ODI, whereas higher scores indicate better status for SF‐36 and ESCA [18–21]. The gait‐related outcome was extracted from the hospital follow‐up form as a pragmatic clinical gait‐related score. Because this score was used in routine clinical follow‐up and was not a formally validated Chinese‐language instrument, it should be interpreted as a supporting functional indicator rather than a standardized patient‐reported measure. Its interpretation was informed by contemporary Japanese spine literature addressing gait disturbance within the Japanese Orthopaedic Association Back Pain Evaluation Questionnaire (JOABPEQ) [22].

### 2.5. Statistical Analysis

All analyses were performed using IBM SPSS 27.0 and cross‐checked against the source dataset. Continuous variables are presented as mean ± standard deviation and categorical variables as number (percentage). Approximate normality of baseline continuous variables was assessed using the Shapiro–Wilk test together with visual inspection of distributions; baseline between‐group comparisons were performed using independent‐samples *t* tests or χ^2^ tests, as appropriate. Longitudinal differences across baseline, 1, 2, and 3 months of follow‐up were examined using generalized estimating equations with an exchangeable working correlation structure and robust standard errors. Each model included group, time, and group‐by‐time interaction terms and was adjusted for age, sex, length of hospital stay, follow‐up duration, and the corresponding baseline outcome score. To estimate adjusted differences at 3 months, multivariable linear regression models were fitted using the same covariates. Sensitivity analyses included reestimation of the 3‐month models with HC3 robust standard errors and a propensity score‐matched analysis. Two‐sided *p* values < 0.05 were considered statistically significant, and 95% confidence intervals are reported. Because all eligible records within the predefined study period were included, no a priori sample size calculation was performed.

## 3. Results

### 3.1. Baseline Characteristics

**FIGURE 1:**
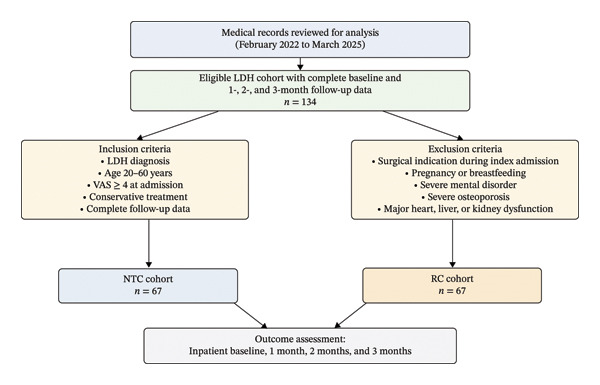
Analytic cohort formation and outcome assessment schedule. Patients meeting the predefined eligibility criteria were classified according to the documented postdischarge nursing pathway into the NTC cohort (*n* = 67) or the RC cohort (*n* = 67). Outcome measures were assessed at inpatient baseline and at 1‐, 2‐, and 3‐month follow‐ups. LDH, lumbar disc herniation; NTC, nurse‐led transitional care; RC, routine care.

A total of 134 eligible records were analyzed, including 67 in the NTC cohort and 67 in the RC cohort. Baseline age, sex distribution, length of hospital stay, and follow‐up duration were similar between groups (Table 2). Baseline VAS, ODI, ESCA, and GS scores were also comparable, whereas the difference in baseline SF‐36 approached but did not reach statistical significance (63.48 ± 2.08 vs 64.15 ± 1.99, *p* = 0.058). Disease duration was reviewed during data extraction, but the retrospective records did not provide sufficiently standardized and complete documentation to support reliable coding for adjusted analysis across the full cohort. To avoid introducing additional misclassification, this variable was not forced into the multivariable models. The formation of the analytic cohorts is illustrated in Figure 1.

**TABLE 2:** Baseline characteristics of the study cohorts.

Variable	NTC (*n* = 67)	RC (*n* = 67)	*t*/*χ* ^2^	*p* value
Age, years	40.36 ± 7.40	40.25 ± 7.14	0.083	0.934
Sex, *n* (%)			0.031	0.860
Male	40 (59.7%)	41 (61.2%)		
Female	27 (40.3%)	26 (38.8%)		
Length of hospital stay, days	7.91 ± 1.33	8.07 ± 1.36	−0.705	0.482
Follow‐up duration, months	10.72 ± 1.59	10.75 ± 1.61	−0.108	0.914
Baseline				
VAS	7.67 ± 0.82	7.63 ± 0.81	0.317	0.752
ODI	37.00 ± 6.03	36.91 ± 5.89	0.087	0.931
SF‐36	63.48 ± 2.08	64.15 ± 1.99	−1.909	0.058
ESCA	36.75 ± 1.34	36.94 ± 1.36	−0.832	0.407
GS	13.16 ± 1.42	13.28 ± 1.38	−0.494	0.622

*Note:* NTC, nurse‐led transitional care; SF‐36, 36‐Item Short Form Health Survey; ESCA, Exercise of Self‐Care Agency scale; GS, gait‐related score. Data are presented as mean ± SD or *n* (%). Percentages are calculated within each cohort. Follow‐up duration refers to the interval from index admission to the last available documented contact in the record system, rather than the prespecified 3‐month outcome assessment window.

Abbreviations: ODI, Oswestry Disability Index; RC, routine care; VAS, visual analog scale.

### 3.2. Propensity Score‐Matched Analysis

**FIGURE 2:**
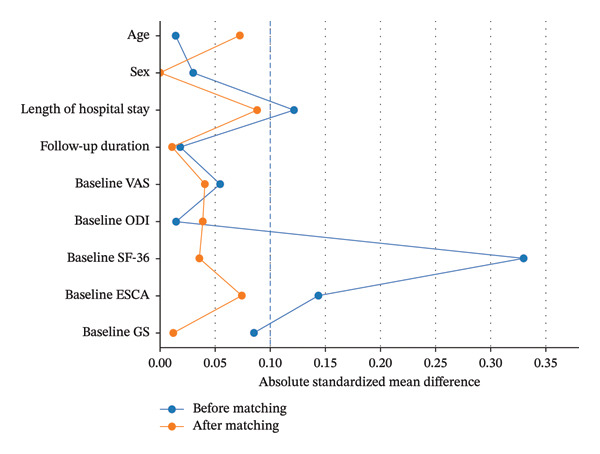
Love plot of absolute standardized mean differences before and after propensity score matching. The dashed reference line at 0.10 indicates acceptable covariate balance after matching.

To assess the robustness of the primary findings, a propensity score‐matched analysis was performed. Using 1:1 nearest‐neighbor matching without replacement, 59 matched pairs were retained for analysis. After matching, baseline covariates were well balanced, as shown in Figure 2, and all absolute standardized mean differences were below 0.10. In the matched cohort, the overall longitudinal pattern remained consistent with the primary analysis: The NTC cohort continued to show lower VAS and ODI scores and higher SF‐36, ESCA, and GS scores during follow‐up. In matched GEE analyses, the group‐by‐time interaction remained significant for VAS (*p* = 0.0366), ODI (*p* = 0.00354), and for SF‐36, ESCA, and GS (all *p* < 0.001).

### 3.3. Pain and Functional Recovery

**FIGURE 3:**
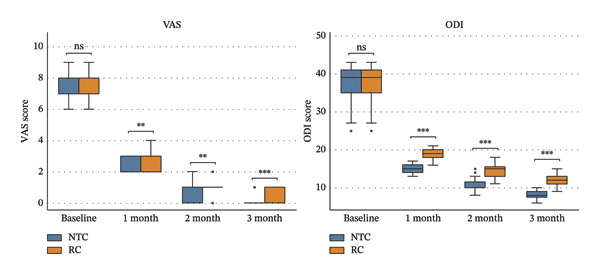
Box plots of VAS and ODI scores in the NTC and RC groups from inpatient baseline to 3‐month follow‐up. VAS ranges from 0 to 10, with higher scores indicating more severe pain. ODI ranges from 0 to 100, with higher scores indicating greater disability. IP, inpatient baseline; FU, follow‐up. Box plots show the median, interquartile range, range, and outliers. Ns, not significant; ^∗^ *p* < 0.05; ^∗∗^ *p* < 0.01; ^∗∗∗^ *p* < 0.001.

Baseline VAS and ODI scores were similar between groups (VAS: 7.67 ± 0.82 vs 7.63 ± 0.81, *p* = 0.752; ODI: 37.00 ± 6.03 vs 36.91 ± 5.89, *p* = 0.931). During follow‐up, the NTC cohort had lower VAS scores than the RC cohort at 1 month (2.34 ± 0.48 vs 2.60 ± 0.55, *p* = 0.005), 2 months (0.64 ± 0.54 vs 0.93 ± 0.56, *p* = 0.003), and 3 months (0.04 ± 0.21 vs 0.46 ± 0.50, *p* < 0.001). ODI values were likewise lower in the NTC cohort at 1 month (15.22 ± 1.17 vs 18.70 ± 1.44, *p* < 0.001), 2 months (10.55 ± 1.38 vs 14.45 ± 1.51, *p* < 0.001), and 3 months (8.37 ± 1.10 vs 11.97 ± 1.30, *p* < 0.001). In adjusted longitudinal analyses, the group‐by‐time interaction was significant for both VAS (GEE interaction *p* = 0.012) and ODI (GEE interaction *p* < 0.001), indicating greater improvement over time in the NTC cohort. At 3 months, multivariable models remained favorable to the NTC cohort for VAS (*β* = −0.42, 95% CI −0.55 to −0.29, *p* < 0.001) and ODI (*β* = −3.62, 95% CI −4.02 to −3.22, *p* < 0.001). Unadjusted distributions for VAS and ODI are shown in Figure 3.

### 3.4. Quality of Life, Self‐Care Ability, and Gait Function

**FIGURE 4:**
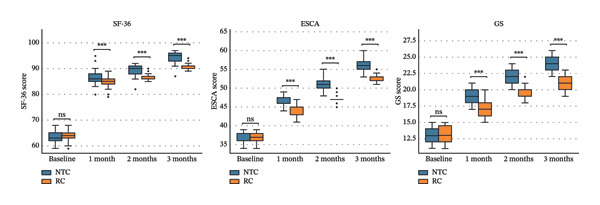
Box plots of SF‐36, ESCA, and GS scores in the NTC and RC groups from inpatient baseline to 3‐month follow‐up. Higher scores indicate better health‐related quality of life (SF‐36), self‐care ability (ESCA), and gait‐related performance (GS). IP, inpatient baseline; FU, follow‐up. Box plots show the median, interquartile range, range, and outliers. Ns, not significant; ^∗^ *p* < 0.05; ^∗∗^ *p* < 0.01; ^∗∗∗^ *p* < 0.001.

Baseline SF‐36, ESCA, and GS scores were similar between groups (all *p* > 0.05). At 1, 2, and 3 months, the NTC cohort showed higher SF‐36 scores (86.51 ± 2.79 vs 84.81 ± 1.94; 89.64 ± 2.18 vs 86.72 ± 1.15; 94.64 ± 2.18 vs 90.72 ± 1.15), higher ESCA scores (46.66 ± 1.26 vs 43.70 ± 1.69; 50.90 ± 1.34 vs 47.03 ± 0.85; 55.90 ± 1.34 vs 52.61 ± 0.80), and higher GS scores (19.16 ± 1.42 vs 17.27 ± 1.11; 22.16 ± 1.42 vs 19.61 ± 1.04; 24.16 ± 1.42 vs 21.03 ± 1.18) than the RC cohort (all *p* < 0.001). Significant group‐by‐time interactions were observed for SF‐36, ESCA, and GS (all *p* < 0.001), indicating greater gains over time in the NTC cohort. Adjusted 3‐month regression models showed the same pattern for SF‐36 (*β* = 4.09, 95% CI 3.51 to 4.68), ESCA (*β* = 3.35, 95% CI 3.02 to 3.69), and GS (*β* = 3.16, 95% CI 2.75 to 3.57) (all *p* < 0.001). These adjusted findings were directionally consistent with the unadjusted comparisons (Table 3), and the unadjusted distributions are shown in Figure 4.

**TABLE 3:** Adjusted, sensitivity, and propensity score‐matched analyses of rehabilitation outcomes.

Outcome	Adjusted 3‐month difference, *β* (95% CI)	*p* value	GEE group‐by‐time *p*	Sensitivity model, *β* (95% CI)	PSM matched GEE group‐by‐time *p*
VAS	−0.42 (−0.55 to −0.29)	< 0.001	0.012	−0.42 (−0.55 to −0.29)	0.0366
ODI	−3.62 (−4.02 to −3.22)	< 0.001	< 0.001	−3.62 (−4.02 to −3.21)	0.00354
SF‐36	4.09 (3.51 to 4.68)	< 0.001	< 0.001	4.09 (3.53 to 4.66)	< 0.001
ESCA	3.35 (3.02 to 3.69)	< 0.001	< 0.001	3.36 (3.02 to 3.69)	< 0.001
GS	3.16 (2.75 to 3.57)	< 0.001	< 0.001	3.16 (2.75 to 3.58)	< 0.001

In this retrospective cohort, patients whose records documented receipt of NTC had better short‐term rehabilitation outcomes than those who received RC alone. These between‐group differences were evident in unadjusted comparisons and persisted in adjusted longitudinal and 3‐month endpoint analyses, suggesting that the observed associations were not fully explained by measured baseline demographic factors or baseline outcome levels.

Several features of the NTC model may help explain these findings. Transitional care extends support beyond hospital discharge, when fragmented services and inconsistent follow‐up may undermine recovery [7, 11, 12]. Programs that combine discharge planning, early nurse contact, and ongoing home‐ or community‐based reinforcement may strengthen continuity, facilitate timely identification of problems, and support adherence to self‐management behaviors [6, 8–10]. Within the NTC pathway, home‐exercise adherence was reinforced during follow‐up calls primarily through patient self‐report and review of exercise logbooks when available; accordingly, adherence support was pragmatic rather than based on a standardized adherence instrument. In LDH, conservative recovery also depends on patient education, exercise implementation, prognostic stratification, and continuity of rehabilitation advice [13, 16, 17]. The greater gains observed in SF‐36, ESCA, and GS are therefore consistent with the possibility that continuity of care influences broader functional adaptation and recovery trajectories in lumbar disc disease [14].

From a clinical perspective, these findings support the relevance of a structured nurse‐led postdischarge pathway for patients with LDH. At the same time, the results should be interpreted as observational associations rather than proof of effectiveness. The adjusted 3‐month ODI difference of 3.62 points, although statistically robust, appears smaller than commonly cited ODI minimum clinically important difference thresholds in lumbar spine populations, suggesting a modest early functional benefit rather than a clearly large clinically important difference [23]. Because the care model was determined in routine practice rather than through random assignment, residual differences in disease severity, rehabilitation adherence, social support, health literacy, or socioeconomic context may still have contributed to the observed outcomes. The adjusted, sensitivity, and propensity score‐matched analyses strengthen the robustness of the findings, but they do not eliminate the possibility of unmeasured confounding.

### 3.5. Limitations

Several limitations should be acknowledged. First, the retrospective single‐center design limits causal inference and may reduce generalizability. Second, cohort classification was based on documented care pathways, and the available records did not permit detailed reconstruction of why some patients received NTC, whereas others received RC, leaving room for selection bias. Although a propensity score‐matched sensitivity analysis yielded directionally consistent results, residual confounding remains possible because allocation‐related factors and several clinically relevant variables were not captured with sufficient completeness and standardization in the retrospective records. Third, although we adjusted for age, sex, length of hospital stay, follow‐up duration, and baseline outcome values, disease duration and symptom chronicity could not be reliably coded from the retrospective records and therefore were not included in the adjusted models. In addition, comorbidities, socioeconomic status, and rehabilitation adherence were not documented in a sufficiently standardized manner to support reliable adjustment. Rehabilitation adherence within the NTC pathway was reinforced through patient self‐report during follow‐up calls and review of exercise logbooks when available, but adherence was not collected as a standardized standalone outcome; self‐reported adherence therefore remains subject to reporting bias. Fourth, the gait‐related score was a pragmatic clinical indicator extracted from the hospital follow‐up form rather than a formally validated Chinese‐language instrument, and it should be interpreted cautiously. Fifth, follow‐up was limited to 3 months. Finally, despite directionally consistent sensitivity analyses, the sample size remained modest for subgroup exploration. Prospective multicenter studies with clearer allocation procedures and longer follow‐up are warranted.

## 4. Conclusions

In this retrospective cohort study, documented receipt of NTC was associated with better pain, disability, quality of life, self‐care, and gait outcomes during the first 3 months after discharge among patients with LDH. These findings support the clinical relevance of structured transitional support while underscoring the need for prospective studies designed to address selection bias and residual confounding.

## Author Contributions

J.G.: primary writing, revision of the manuscript, study concept and design, statistical support, and critical revision of the manuscript. J.L.: study concept and design, literature screening, and desk review. J.C.: initial writing and revision of the manuscript. P.L.: literature screening, data extraction, statistical analysis, and revision of the manuscript. H.W.: critical revision of included studies and statistical support. All authors have made substantive contributions to this study and manuscript, and all have reviewed the final paper before its submission.

## Funding

This research received no specific grant from any funding agency in the public, commercial, or not‐for‐profit sectors.

## Ethics Statement

This retrospective study was conducted in accordance with the ethical standards of the Declaration of Helsinki and approved by the Ethical Review Board of Hainan District People’s Hospital (Approval No. 1503032021003). All patient data were deidentified prior to analysis, and the requirement for individual informed consent was waived.

## Conflicts of Interest

The authors declare no conflicts of interest.

## Data Availability

The data supporting the findings of this study are not publicly available because they were derived from deidentified clinical records and follow‐up data from a single institution and may contain information that could compromise participant privacy. The data are available from the corresponding author upon reasonable request, subject to approval by the relevant institutional and ethical authorities.
